# Long-term follow-up of nephrotoxicity in rats administered both melamine and cyanuric acid

**DOI:** 10.1186/1756-0500-7-87

**Published:** 2014-02-08

**Authors:** Takahiro Yasui, Takahiro Kobayashi, Atsushi Okada, Shuzo Hamamoto, Masahito Hirose, Kentaro Mizuno, Yasue Kubota, Yukihiro Umemoto, Noriyasu Kawai, Keiichi Tozawa, Bing Gao, Kenjiro Kohri

**Affiliations:** 1 Department of Nephro-urology, Nagoya City University Graduate School of Medical Sciences, 1 Kawasumi, Mizuho-cho, Mizuho-ku, Nagoya 467-8601, Japan; 2 China-Japan Kidney Stone Research Center, Shenyang, China; 3 School of Basic Medical Sciences, Shenyang Medical College, Shenyang, China

**Keywords:** Melamine, Cyanuric acid, Renal failure, Renal calculi

## Abstract

**Background:**

Melamine was recently identified as a risk factor for renal calculi following the milk powder contamination in China. However, the long-term natural history of melamine exposure and its renal effects remain unknown. We evaluated renal function and other adverse health effects using a rat model administered melamine and cyanuric aid, considering age and sex.

**Methods:**

Twelve male F334/N rats each of ages 6, 10, and 26 weeks (N = 36) were equally assigned to Group M + C or controls. Group M + C rats were administered 12 mg · kg^-1^ · day^-1^ of melamine and cyanuric acid for 28 days. Serum and urine samples and kidney sections were evaluated on day 28. Six-week-old male and female F344/N rats were administered 12 mg of melamine and cyanuric acid for 28 days. Body weights were measured weekly; on days 0, 28, 90, and 180 after the 28-day period of melamine and cyanuric acid administration, serum samples and kidney sections were obtained.

**Results:**

Although the control group had no crystals, 6-week-old Group M + C rats had more crystals compared to the 10- and 26-week old Group M + C rats. Male rats also had significantly more crystals than females of the same age. Male rats were affected to a greater extent than females.

**Conclusion:**

Younger rats experienced more severe renal failure and greater renal crystal deposition following melamine and cyanuric acid administration. However, after melamine and cyanuric acid administration cessation, crystal deposition and renal failure improved and did not cause growth arrest. Therefore, early diagnosis of melamine-associated calculi is critical.

## Background

Urinary calculi rarely occur in infants. However, since September 2008, an unusually high incidence of urinary calculi in infants was reported in China. It is believed that consumption of melamine-contaminated powder formula led to this epidemic of urinary calculi among infants [1]. Melamine (1,3,5-triazine-2,4,6-triamine) is an industrial chemical compound that has a high amount of non-protein nitrogen. It is produced on a large scale in the United States and other countries. For example, China’s production of melamine accounts for more than 50% of the total output in Asia and represents almost one-quarter of the global production. Melamine resins are manufactured from melanin and used as raw materials for a wide variety of products, including laminates, adhesives molding compounds textiles, and flame retardants [2,3].

In the United States, melamine was recently implicated in a foodborne outbreak of renal disease among domestic cats and dogs [4-6]. The commercial pet food had been adulterated with melamine to augment the protein content of pet food. In the 2008 outbreak of urinary calculi in Chinese infants, melamine had been added to milk for human consumption in order to achieve satisfactory protein concentrations in China [7]. Furthermore, reported cases of renal calculi and acute renal failure have continued to increase since 2008 among Chinese children fed infant formula contaminated with melamine. According to a 2008 WHO report, 294,000 infants were symptomatic, resulting in 51,900 hospitalizations and 6 deaths from renal failure [7-9]. Accordingly, public concern over food safety has understandably escalated to new heights.

Although the nephrotoxic effects observed in animals that ingested melamine-contaminated pet food involved calculi consisting of both melamine and cyanuric acid, the toxic effects observed in infants who ingested contaminated milk powder appear to be due to melamine alone. The concomitant presence of cyanuric acid does not seem to be necessary to produce melamine-associated adverse effects in humans. In a previous study, we reported that melamine and cyanuric acid in combination, but not melamine alone, induced crystal formation and affected renal function [10]. Moreover, we found that renal failure due to melamine-cyanurate crystals appeared to occur via tubular occlusion using X-ray diffraction analysis with scanning electron microscopy (SEM) [10].

According to the Chinese Ministry of Health’s standards of diagnosis and treatment, infants with melamine-associated urinary calculi typically pass the smaller stones spontaneously and conservative treatment is generally sufficient in such cases. In addition, when nephrolithiasis caused by melamine is discovered, administration of milk containing melamine should immediately be discontinued.

However, the long-term natural history of co-exposure of melamine and cyanuric acid, and its renal effects on growth remains unknown. Therefore, in this study, we aimed to examine renal function and other adverse health effects using a rat model administered melamine and cyanuric acid, taking into account age and sex.

## Methods

### Experimental protocol

First, we examined the effects of melamine and cyanuric acid administration according to age. Twelve male F334/N rats each of ages 6, 10, and 26 weeks (N = 36, Japan SLC. Inc. Shizuoka, Japan) were assigned in equal numbers to either the melamine and cyanuric acid administration group (Group M + C) or control group, such that there were 6 rats of each age in both groups. The 18 control rats were administrated 1 mL of water daily, whereas the 18 Group M + C rats were administered melamine and cyanuric acid at a dose of 12 mg · kg^-1^ · day^-1^, via a stomach tube for 2 weeks for each age group. Melamine and cyanuric acid (Sigma-Aldrich, Milwaukee, USA) were mixed with water at room temperature. Serum samples, 24-h urine samples, and kidney sections were obtained on day 28 from all the rats (6 rats in each group).

Second, we considered the effects of melamine and cyanuric acid administration according to sex. Twelve 6-week old male and female F344/N rats (N = 24, Japan SLC) were divided into groups based on sex. Rats were administered melamine and cyanuric acid at a dose of 12 mg · kg^-1^ · day^-1^ for 28 days via stomach tubes, as described above. Body weights were measured every week. Serum samples, 24-h urine samples, and kidney sections were obtained on days 0, 28, 90, and 180 after the 28-day period of melamine and cyanuric acid administration.

The animals were housed individually in plastic shoebox cages with wood shavings. All experimental procedures were performed in accordance with the NIH Guide for the Care and Use of Laboratory Animals and approved by both the Animal Care and Use Committee and Biological Safety Committee of Nagoya City University.

### Blood chemistry and urine variables

Blood biochemistry and urine calcium, phosphate, creatinine, and magnesium level measurements were conducted by Mitsubishi Chemical Medicine Corporation (Tokyo, Japan). Urinary pH and urine volumes were measured manually.

### Urinary sediments

Urine was aspirated from the bladder using a 22-G needle during euthanasia on day 28 of administration, and centrifuged at 1,500 rpm for 10 min at room temperature. Urine volume collection was performed by using a special cage (KN-646, Natsume Factory, Tokyo, Japan) that was developed to avoid contamination. Crystals were observed through light microscopy and polarized light optical microphotography.

### Kidney tissue processing

After the rats were killed, we performed histopathological analysis on the kidney samples. The resected kidney specimens were immediately fixed in 4% paraformaldehyde and embedded in paraffin. Four-um-thick cross sections ware stained with hematoxylin and eosin (HE), Oil Red O (a stain for lipid and plastics), and von Kossa (a statin for phosphates). To confirm that the stained materials were crystals for each staining method, sections were observed using polarized light optical microphotography. Crystal formation was assessed quantitatively using the NIH Image 1.61 software (Scion Inc., Bethesda, USA).

The pathological severity of renal injury has been graded according to the criteria described by Shackelfold et al. [11]. Lesions were graded from 1 to 5 according to the severity: 1, minimal (< 1%); 2, slight (1–25%); 3, moderate (26–50%); 4, moderate/severe (51–75%); and 5, severe/high (76–100%). To compare the severity of lesions in different groups according to the semi-quantitative renal injury score, the mean scores (calculated by dividing the sum of the score per grade of affected rats by the total number of examined rats; expressed as mean and standard deviation) were used for further analysis.

### Microstructural observation via scanning electron microscope

One kidney from each rat was processed for analysis with optical microscopy, whereas the other was processed for analysis with the SEM. In the latter case, the tissue samples were cut into 4-um sections and submerged in a phosphoric acid buffer. They were then re-fixed, first using tetroxide. A 50–100% ethanol series was used to dehydrate the samples. The samples were then embedded in epoxy resin, coated with platinum, and photographed using SEM (S-4800, Hitachi, Tokyo, Japan).

### Statistical analysis

Data are presented as the mean ± SD. Groups were compared using the Mann–Whitney *U* test, with P < 0.05 considered statistically significant.

## Results

### Crystal formation following melamine and cyanuric acid according to age

Serum levels and urinary biochemical findings according to age in male rats 28 days after administration of melamine and cyanuric acid are shown in Table 1. There were no significant differences between Group M + C and the controls, with the exception of serum creatinine, blood urea nitrogen, and urine volume. Compared to the controls, Group M + C had a significantly higher serum creatinine at all ages, particularly in 6-week-old rats. Moreover, Group M + C had a significantly higher blood urea nitrogen in 6-week-old rats and 10-week-old rats. In addition, these 6-week-old rats in Group M + C had significantly greater urine volumes compared to the controls (P < 0.05). The urine volumes of 10- and 26-week-old rats from Group M + C were also higher than those of the controls, although this difference did not reach statistical significance. The renal injury in Group M + C was significantly more severe compared to that of the controls. The 6-week-old rats in Group M + C exhibited a more severe renal injury compared to that of 10-week-old rats. Moreover, 26-week-old rats in Group M + C had a less severe renal injury compared to that of 6-week-old and 10-week-old rats.

**Table 1:** Serum and urinary biochemical findings

**Age**	**6 weeks old**		**10 weeks old**		**26 weeks old**	
**Group**	**Control**	**Group M + C**	**Control**	**Group M + C**	**Control**	**Group M + C**
Serum Cr (mg/dL)	0.23 ± 0.05	0.68 ± 0.27*	0.25 ± 0.04	0.55 ± 0.24*	0.32 ± 0.08	0.45 ± 0.20*
Serum BUN (mg/dL)	18.8 ± 1.66	61.4 ± 28.4*	19.1 ± 1.84	52.4 ± 24.8*	28.8 ± 5.11	30.8 ± 11.8
Seruma Ca (mg/dL)	9.57 ± 0.12	9.55 ± 0.19	9.04 ± 0.23	9.56 ± 0.33	9.34 ± 0.24	9.44 ± 0.35
Serum P (mg/dL)	9.02 ± 0.25	8.02 ± 0.93	8.91 ± 0.30	8.61 ± 0.88	8.75 ± 0.28	8.81 ± 0.48
Urine volume (mL)	19.3 ± 3.4	82.2 ± 33.9*	21.3 ± 8.3	67.3 ± 30.3	38.3 ± 13.3	48.3 ± 23.3
Urine pH	7.60 ± 0.45	8.00 ± 0.40	7.40 ± 0.50	7.60 ± 0.45	7.50 ± 0.50	7.55 ± 0.60
Urine Ca (mmol/g/Cr)	0.06 ± 0.03	0.19 ± 0.12	0.06 ± 0.08	0.10 ± 0.18	0.10 ± 0.12	0.08 ± 0.12
Urine P (mmol/g/Cr)	3.16 ± 1.07	2.13 ± 2.36	3.09 ± 1.03	2.98 ± 2.01	3.12 ± 1.13	3.07 ± 2.03
Urine Mg (mmol/g/Cr)	0.17 ± 0.15	0.78 ± 1.15	0.15 ± 0.22	0.23 ± 0.52	0.18 ± 0.14	0.15 ± 0.34
Pathological severity of renal injury	0.17 ± 0.41	4.17 ± 0.75*	0.33 ± 0.52	3.83 ± 0.75*	0.83 ± 0.75	2.66 ± 0.52*

Serum levels, urinary biochemical findings, and pathological severity of renal injury according to age in male rats at 28 days after melamine and cyanuric aid administration compared to the control rats.

Data are presented as the mean ± SD.

Abbreviations: *Cr* creatinine; *BUN* blood urea nitrogen; *Ca* calcium; *P* phosphorus; *Mg* magnesium.

*P < 0.05, versus the control group.

**Figure 1:**
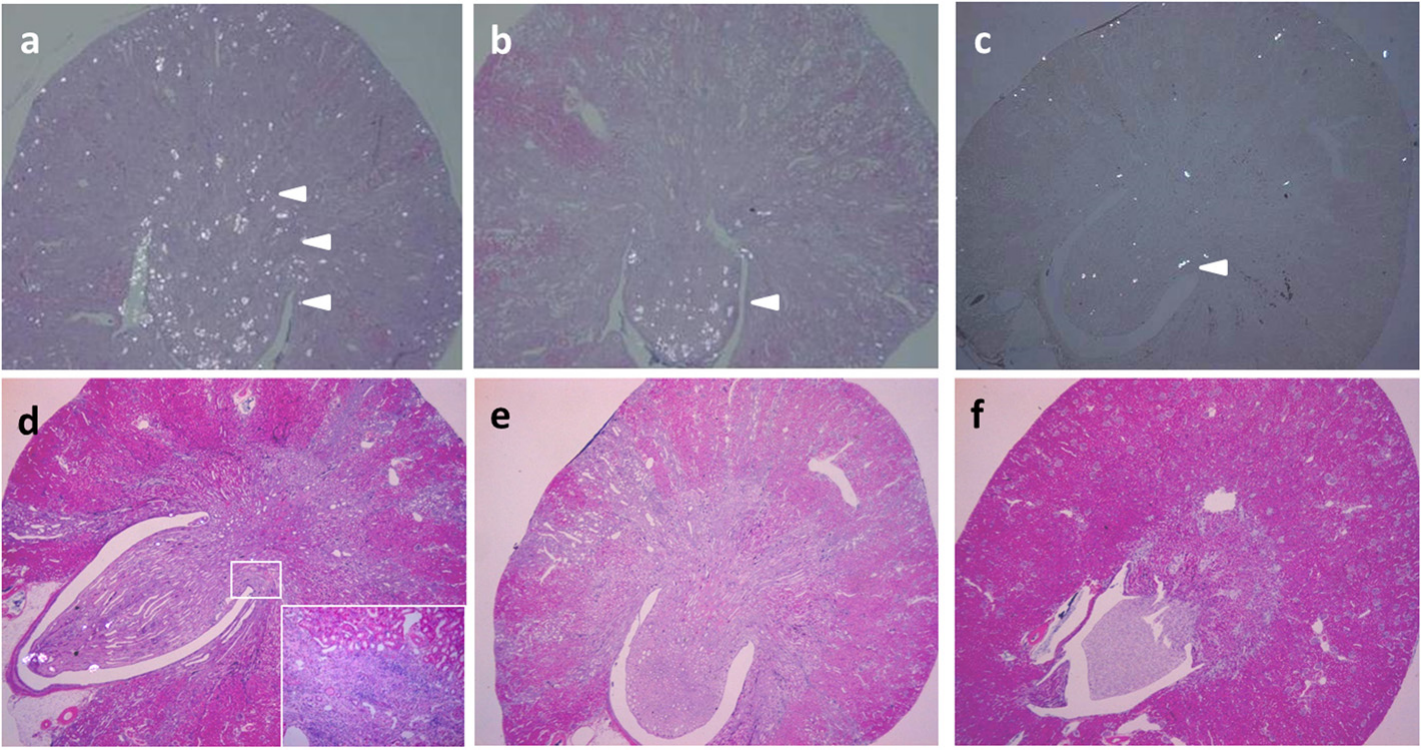
**Microscopic appearance of kidney sections according to age in Group M + C Crystal distributions are observed using polarized light optical microphotography (a-c), and scarring was noted with HE staining (d-f) of the paraffin-embedded axial sections in Group-M + C at day 28 in rats aged: 6 weeks (a, d), 10 weeks (b, e), and 26 weeks (c, f) (×20).** Arrowheads indicate crystals, and arrows indicate scarring. Inset in **(d)** reveals the kidney scarring (×190).

**Figure 2:**
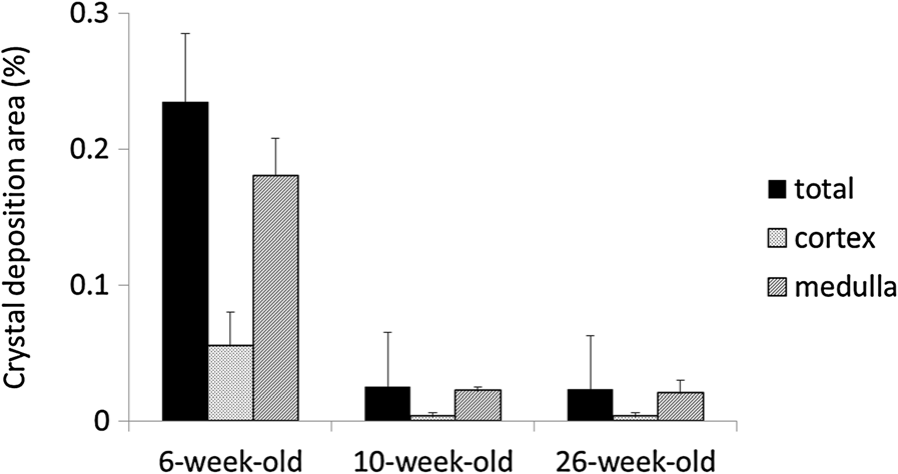
**Area of deposition of melamine and cyanuric acid renal crystals according to age (total deposition area divided by the area of the cortex and medulla).** There were no crystals in the control group. *P < 0.05, comparison with 6-week-old rats.

Renal crystals were observed using polarized light optical microphotography (Figure 1a-c). However, crystals were not found at any age in the control group. In Group M + C, there were more crystals in the 6-week-old rats compared to those in the 10- and 26-week-old rats (Figure 2). Crystal formation was noted in all areas of the kidney, including the cortex and medulla, in the 6-week-old rats; however, crystal formation was mostly limited to the medulla in the 10- and 26-week-old rats. This crystal formation may have caused the increase in serum creatinine and blood urea nitrogen. In particular, these 10- and 26-week-old rats had only slightly formed crystals. This finding suggests that crystal formation occurs readily in young male rats following administration of melamine and cyanuric acid.

**Figure 3:**
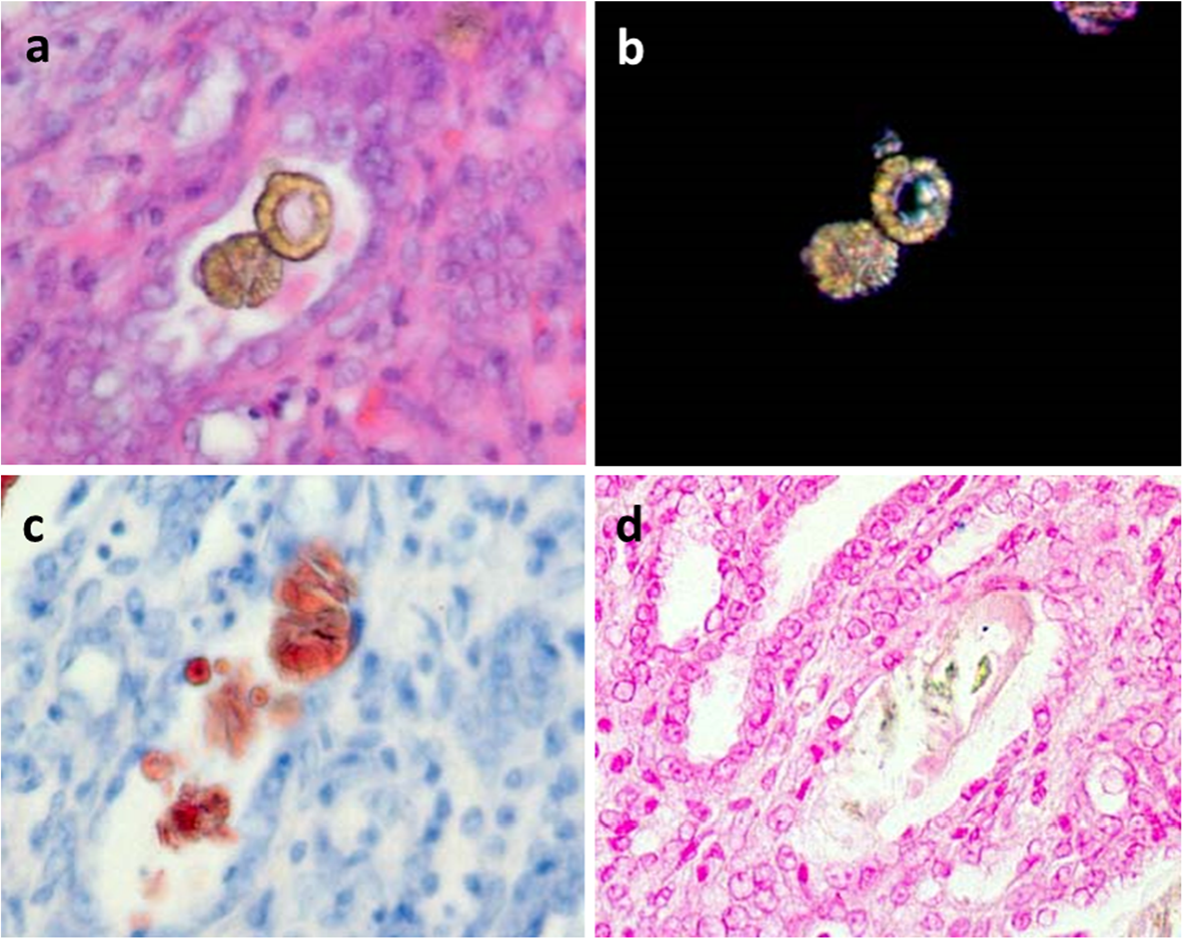
Characteristics of crystals from the distal tubules of 6-week-old rats from Group M + C are observed as light brown staining on HE staining (a), polarized light optical microphotography (b), red with axle-shaped layers on Oil Red O staining (c), and lack of staining on von Kossa staining (d) (×200).

Consecutive kidney sections taken from the 6-week-old rats of Group M + C were stained with HE (Figure 3a), polarized light optical microphotography (Figure 3b), Oil Red O staining (Figure 3c), and von Kossa staining (Figure 3d), revealing the characteristics of crystals from the proximal tubules. The crystals manifested as light brown on HE staining and red with axle-shaped layers on ORO staining. On von Kossa staining, the crystals were not detected. Crystals can be detected on polarized light optical microphotography. ORO staining and von Kossa staining primarily detect fat and calcium phosphate crystals, respectively. The characteristics of crystals formed in the Group M + C rats—such as brown color on HE staining, birefringence under polarized light optical microphotography, lack of staining with von Kossa, and red color on ORO staining—are similar to those of melamine cyanurate crystals.

Furthermore, the kidneys of the 6-week-old rats from Group M + C had scarring and fibrosis; however, this was not observed in the 10- and 26-week-old rats from Group M + C (Figure 1d-f).

### The changes noted after exposure to melamine and cyanuric acid

**Figure 4:**
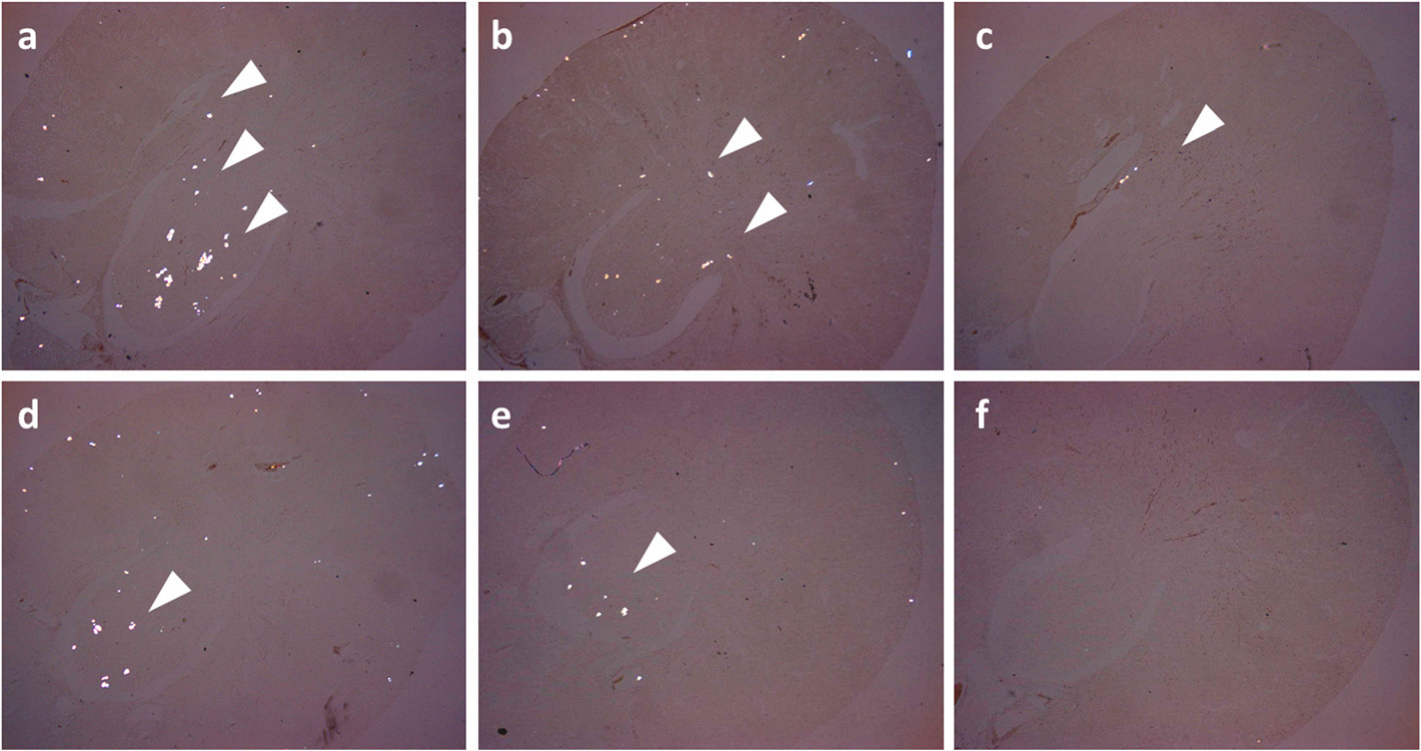
**Change in crystals after melamine and cyanuric acid administration in 6-week-old male rats (a-c) and female rats (d-f) observed using polarized light optical microphotography (×40).** Crystals are seen at day 0 **(a, d)**, day 14 **(b, e)**, and day 28 following the 28-day administration period **(c, f)**. Arrowheads indicate crystals.

**Figure 5:**
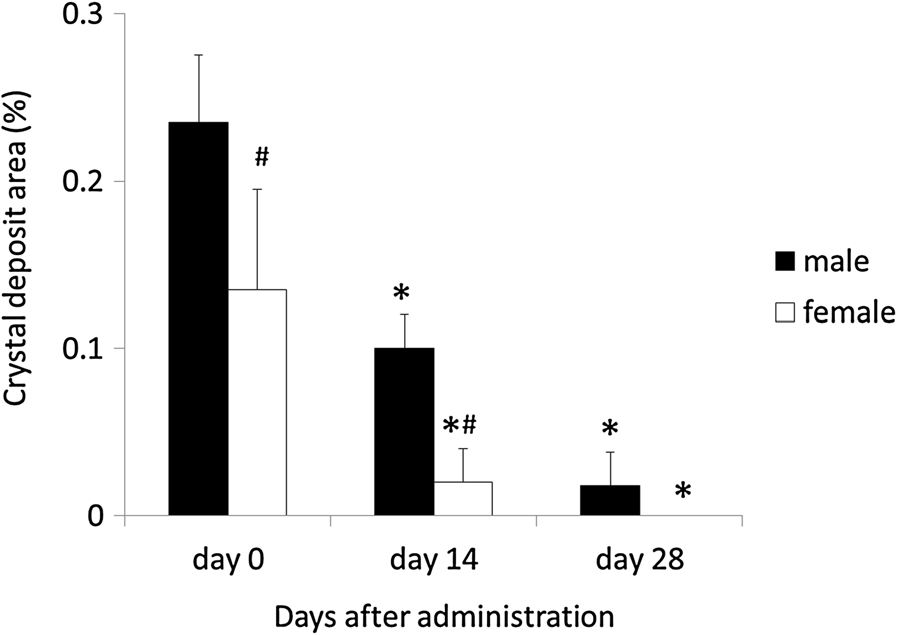
**Change in crystal deposition areas after administration of melamine and cyanuric acid in 6-week-old rats.** *P < 0.05 comparison with males. ^#^P < 0.05 comparison with day 0.

For comparison between the sexes, 6-week-old rats were chosen as they exhibited a greater amount of crystal formation and more severe renal failure as compared to rats at other ages. Melamine and cyanuric acid administration were significantly more likely to cause crystal deposits in males compared to females of the same age (Figure 4a,d and Figure 5). After exposure to melamine and cyanuric acid, renal crystal deposits decreased gradually. In male rats, on day 14 after melamine and cyanuric acid administration, crystal deposits noticeably decreased, and by day 28 after administration, they had almost disappeared (Figure 4b,c and Figure 5). In male rats, the serum creatinine level had also decreased gradually after exposure to melamine and cyanuric acid (0.36 ± 0.06, 0.33 ± 0.08, and 0.28 ± 0.05 mg/dL on day 0, day 14, and day 28 after administration, respectively). In female rats, on day 14 after melamine and cyanuric acid administration, crystal deposits began to appear, and by day 28, there were no crystal deposits observed in the kidney (Figure 4e,f and Figure 5). In female rats, the serum creatinine level had also decreased gradually after exposure to melamine and cyanuric acid (0.24 ± 0.04, 0.24 ± 0.05, and 0.23 ± 0.03 mg/dL on day 0, day 14, and day 28 after administration, respectively).

**Figure 6:**
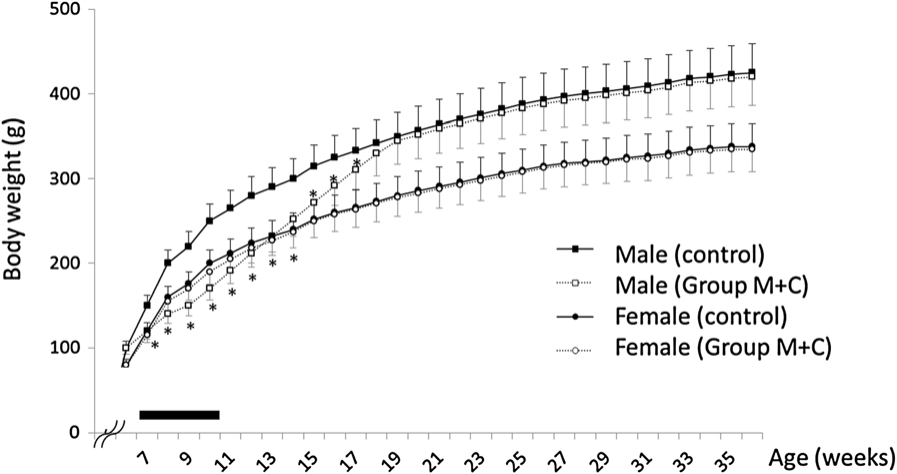
**Change in body weight after administration of melamine and cyanuric acid.** The bar indicates the 28-day period of melamine and cyanuric acid administration in 6-week-old rats. Controls did not receive melamine and cyanuric acid. *P < 0.05, comparison with the controls in male rats.

In this study, we aimed to assess the recovery and growth after exposure to melamine and cyanuric acid, as well as after the disappearance of crystal deposition. Male rats from Group M + C had significantly lower body weights compared to those of the controls during and after administration (Figure 6). After the conclusion of melamine and cyanuric acid administration, body weights gradually recovered. We found that the body weights of male rats, aged ≥18 weeks, in Group M + C were not significantly different compared to those of age-matched controls of the same age. In female rats, the body weights were also not significantly different between Group M + C and controls. Compared to the controls of the same age, female rats of Group M + C tended to have lower body weights. However, after the conclusion of melamine and cyanuric acid administration, the female rats’ body weights gradually caught up to those of the controls.

**Figure 7:**
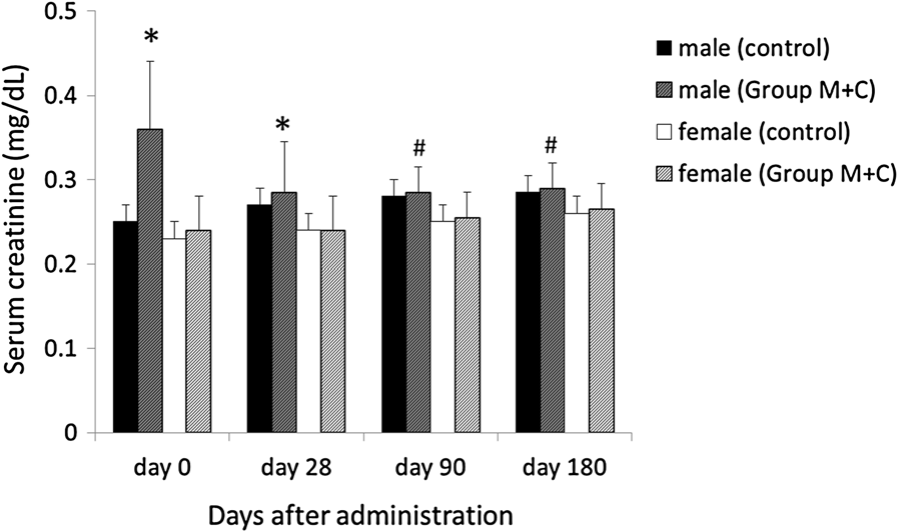
**Change in serum creatinine level after administration of melamine and cyanuric acid in 6-week-old rats.** *P < 0.05, comparison with controls. ^#^P < 0.05, comparison with day 0.

Melamine and cyanuric acid administration led to increased serum creatinine levels in male rats (Figure 7). However, after the conclusion of melamine and cyanuric acid administration, serum creatinine levels decreased, with no significant difference found compared with controls on day 28. On day 90, serum creatinine levels of Group M + C male rats equaled those of the controls.

**Figure 8:**
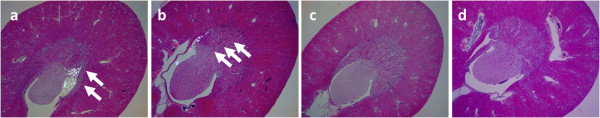
**Change in renal scarring after administration of melamine and cyanuric acid.** Scarring was observed on day 0 **(a)** and day 28 **(b)** after administration; however, it had disappeared by day 90 **(c)** and day 180 **(d)**. (HE. ×40). Arrows indicate scarring of kidney tissue.

Furthermore, after the 28-day period of administration, we observed renal scarring and fibrosis (Figure 8a). On day 28 after the conclusion of administration, the areas of renal scarring and fibrosis appeared to decrease, and on day 90, the scarring and fibrosis were minimal. By day 180, the scarring and fibrosis had disappeared (Figure 8b-d).

## Discussion

In the 2008 China outbreak of urinary calculi among infants, the melamine-tainted milk products had been fraudulently adulterated to augment the apparent protein content [12]. Since melamine is not metabolized and rapidly eliminated in urine, melamine and its structural analogues, including cyanuric acid, may interact to form melamine-cyanurate crystals in cats and dogs [13]. In comparison, human and primates have much higher serum uric acid concentrations than cats and dogs and hence are more likely to form melamine-urate crystals [13]. An animal study found that those fed melamine developed renal calculi, resulting in urinary tract obstruction [14]. Deaths due to urinary tract calculi and acute renal failure have been reported in infants and young children exposed to very high melamine levels for prolonged periods [7-9]. In a previous study, we reported an association between sex and melamine-associated renal calculi, indicating a male-to-female ratio of 2.4:1 in cases with melamine calculi [15]. Similarly, Guen et al. reported a male-to-female ratio of 1.5:1 [7]. The different levels of uric acid and estrogen may account for this lower incidence of melamine-associated renal calculi in females than in males [7].

We have previously reported the mechanism of renal calculi formation and the dose required to induce crystal deposits and renal failure [10]. A combination of melamine and cyanuric acid, but not melamine alone, induces crystal formation and affects renal function [10]. Furthermore, tubular occlusion by melamine-cyanurate crystals appears to cause renal failure [10]. Other studies have reported that the combined administration of melamine and cyanuric acid increases nephrotoxicity [16-19].

In the present study, Group M + C rats exhibited crystals characterized by brown color staining with HE, birefringence under polarized light optical microphotography, lack of staining with von Kossa, and red color staining with Oil Red O, which are similar to those of melamine-cyanurate crystals found in animals that died in 2007 after consuming pet food contaminated with melamine and cyanuric acid [4-6]. Furthermore, an energy-dispersive X-ray analysis in our previous study indicated that the renal crystal components included carbon, nitrogen, and oxygen, but not calcium, whereas those from peripheral tissue included carbon and oxygen [10]. This finding indicates that melamine-cyanurate crystals are completely different from the typical calcium-associated calculi.

This study has 2 significant findings. First, young rats administered melamine and cyanuric acid experience more severe renal failure and greater renal crystal deposits compared to that noted in adult rats. The findings of biochemical examination and pathological examination of renal injury and crystal deposition indicated that young rats are influenced to a greater extent by the administration of melamine and cyanuric acid than adult rats. Furthermore, male rats are also more predisposed to adverse outcomes than females. Second, renal failure caused by melamine-cyanurate crystals can improve after cessation of melamine and cyanuric acid administration, and its severity may be related to the degree of melamine exposure. Hence, younger infants have a greater risk of renal crystal deposits and failure, particularly males. In the present study, young rats (6 weeks old) exhibited crystal formation in the kidney cortex, but this was not noted among older rats (10 and 26 weeks old). This crystal formation in the cortex may induce renal failure. Severe crystal deposition in the collecting duct of the medulla may induce crystal deposition in the cortex, and thus increase nephrotoxicity. Moreover, early diagnosis of melamine exposure and therefore immediate discontinuation of this nephrotoxic agent may prevent and/or minimize associated morbidity and mortality.

A few patients with melamine-associated urolithiasis develop renal failure, usually due to urinary obstruction. These patients’ symptoms may include nausea, vomiting, abdominal distention, fever, hematuria, oliguria, and anuria [20]. A position statement by a committee of pediatric nephrologists suggests that patients with suspected melamine exposure should undergo urinalysis, routine blood investigations, and appropriate imaging [21]. Ultrasonography is the preferred imaging modality to diagnose melamine-associated urolithiasis in children [22,23].

Most patients with melamine-associated renal failure can be successfully managed with conservative management, including intravenous infusion of fluids and urinary alkalization. After the calculi have passed, renal function usually recovers [24,25]. Potassium sodium hydrogen citrate is a promising treatment, as it increases the likelihood of expulsion of melamine-associated calculi [20]. In a study of 25 patients with melamine-associated renal calculi complicated by acute obstructive renal failure, Sun et al. reported that renal function eventually returned to normal in all patients after various durations of therapy [26]. In addition, 90% of the patients expelled all calculi within 6 months and have experienced no sequelae to date. Our study findings in a rat model were similar to the clinical course described, and thus effectively confirmed the natural history of renal function recovery.

## Conclusions

Compared to adult rats, young rats experience more severe renal failure and greater renal crystal deposits following administration of melamine and cyanuric acid. Furthermore, male rats were affected to a greater extent that females. The degree of recovery from renal failure due to crystal deposits may be associated with the levels of melamine exposure. However, after immediate discontinuation of melamine, crystal deposition and renal failure improved and did not appear to cause growth arrest. Therefore, early diagnosis of melamine calculi is critical to minimize morbidity and mortality.

## Abbreviations

SEM: Scanning electron microscopy; HE: Hematoxylin and eosin.

## Competing interests

All authors declare that they have no competing interests.

## Authors’ contributions

Study design: TY and KK; Study conduct: TK, AO, SH and MH; Data collection: KM, YK and YU; Data interpretation: TY, NK, KT and GB; Drafting of the manuscript: TY and KK. All authors have read and approved the final manuscript.
